# Long-Term Results after Early Secondary Repair of Obstetric Anal Sphincter Injury: A Case Series and Literature Review

**DOI:** 10.1007/s00192-025-06081-6

**Published:** 2025-02-20

**Authors:** Iben Onsberg Hansen, Ulla Due, Safia Habes, Karoline Daniel Dynesen, Niels Klarskov, Hanna Jangö

**Affiliations:** 1 Department of Obstetrics and Gynecology, Herlev University Hospital, Borgmester Ib Juuls Vej 1, 16th Floor, 2730 Herlev, Capital Region Denmark; 2 Faculty of Health and Medical Sciences, University of Copenhagen, Copenhagen, Denmark; 3 Department of Physiotherapy and Occupational Therapy, Herlev University Hospital, Herlev, Capital Region Denmark; 4 Department of Gastrointestinal Surgery, Herlev University Hospital, Herlev, Capital Region Denmark; 5 Department of Gastrointestinal Surgery, Nordsjællands Hospital Hillerød, Hillerød, Capital Region Denmark

**Keywords:** OASI, Early secondary repair, Anal incontinence, Long-term outcome, Quality of life, Sexual function

## Abstract

**Introduction and Hypothesis:**

The incidence of obstetric anal sphincter injury (OASI) is 3.6–6% of women with vaginal deliveries. Complications to OASI are common, and secondary repair is needed in 2.6–3%. Traditionally, secondary repair has been postponed until wound healing, but studies have shown that early secondary repair within 21 days can be safely performed.

**Methods:**

The aim of this cohort study and literature review was to investigate the long-term outcomes after early secondary repair with focus on anal incontinence, quality of life and impact on sexual function with the use of International Consultation on Incontinence Questionnaire-Bowel (ICIQ-B).

**Results:**

A total of 17 patients underwent early secondary repair after OASI within the study period and 11 answered and returned the long-term follow-up questionnaire. Seven had no postoperative complications, nine had infection and two developed recto-vaginal fistulas that needed subsequent surgical treatment. Median follow-up period was 5 years (2.3–5.7). At long-term, ten women (91%) reported fecal urgency, nine (82%) flatal and liquid incontinence, six (55%) problems with soiling and six (55%) unpredictable bowel accidents. Five women (45%) planned daily activities to accommodate their anal incontinence and three (27%) stayed at home because of anal incontinence. Seven women (64%) reported restrictions in their sexual relations due to anal incontinence.

**Conclusions:**

In conclusion, early secondary repair of OASI in women with severe wound dehiscence involving the anal sphincter may be necessary. However, this group have a high risk of anal incontinence, negative impact on quality of life, and risk of sexual dysfunction at long-term follow-up.

## Introduction

The incidence of obstetric anal sphincter injury (OASI) ranges widely and is probably underestimated [1]. Approximately 3.6–6% of women with vaginal deliveries sustain an OASI and roughly 85% are first-time mothers [1, 2]. OASI is a well-known risk for anal incontinence (defined as involuntary loss of feces or flatus [3]).

In some countries, the incidence of OASI has been reduced over the years because of preventive strategies using manual techniques to protect the perineum during the second stage of delivery [4]. In Norway, “the Finnish grip”, has been re-introduced for perineal support and along with slowing down the speed of crowning, focus on communication and lateral episiotomy on indication, the risk of OASI has been halved in all vaginal births in Norway [5]. Similar results have been seen in Denmark [6].

In women with OASI, complications are common and 7–20% experience wound infection and/or dehiscence after the primary repair [7, 8]. Thus, secondary repair is needed in 2.6–3% of women with OASI due to wound complications or due to clinically undiagnosed OASI at delivery [7, 9].

Traditionally, secondary repair has been postponed till after primary healing. Late secondary repair is typically performed more than 3 months after delivery, but often several years after, as some women seek healthcare because of symptoms with anal incontinence at a later timepoint. Some studies have shown, that early secondary repair (approximately within 21 days after delivery) can be safely performed with similar long-term outcomes related to anal incontinence [10]. However, data are limited, and it seems that women with early secondary repair have a high risk of complications and quite poor outcome [11, 12].

The aim of this study was (1) to investigate the long-term outcomes after early secondary repair in women with obstetric anal sphincter injury and the related consequences on anal incontinence, quality of life and sexual relations and (2) to perform a systematic review to evaluate risk of postoperative complications and long-term outcome in the literature.

## Materials and Methods

We performed a retrospective cohort study with prospective follow-up of all women with OASI who had received early secondary repair within the first month after delivery.

### Patient Selection

Patients operated with early secondary repair were identified using the NOMESCO classification of Surgical Procedures in combination with The International Classification of Diseases and Related Health Problems, ICD-10. Medical journals were revised to ensure that only women with early secondary repair were included. Data was extracted from the medical journals.

**Fig. 1:**
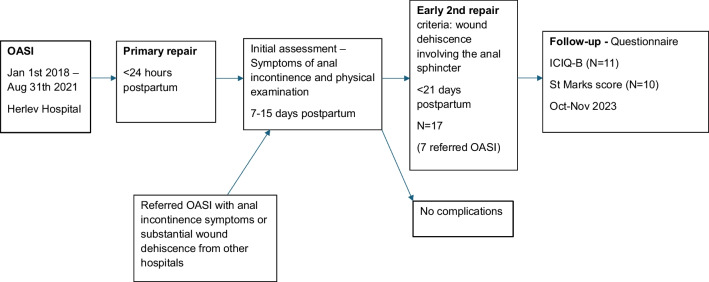
Timeline for women with early secondary repair

We included all patients in the period 01.01.2018–31.08.2021 who had a secondary repair within approximately 30 days after an OASI. The early secondary repairs were all performed at the Department of Obstetrics and Gynecology Herlev University Hospital—a highly specialized center for secondary repair according to The Danish National Health Board classification. See timeline in Fig. 1.

### Secondary Suture Technic and Criteria

The classification of the perineal rupture was performed by the obstetrician who performed the primary repair within 24 h of delivery. The women were offered a control visit 7 to 15 days after the primary repair, including assessment of anal incontinence symptoms and examination of the primary repair. In some cases, endoanal ultrasound was performed to evaluate the suturing and healing process. Only patients with a complete or near-complete rupture of sutures involving the external anal sphincter were offered secondary repair. Surgical technique is standardized and performed by a member of the OASI-team (urogynecologists) at the operating theater under general or spinal anesthesia. Broad-spectrum intravenous antibiotics were administered to all patients during the procedure. Primary repair was demolished as well as removal of necrotic and granulation tissue before external and internal anal sphincter was repaired separately using 2–0 Vicryl end-to-end interrupted sutures. The superficial transverse perineal muscle and the bulbocavernosus were identified and repaired separately. The vaginal epithelium and the perineal skin were closed with running sutures. If the anal epithelium was involved, it was repaired separately using a running absorbable suture. The repair was in all cases done without a covering stoma.

All postoperative complications were assessed by reviewing medical records. The diagnosis of fistulas was based on symptoms and characteristic findings on physical examination in the outpatient urogynecological clinic.

### Questionnaire

All patients received a validated Danish version of the internationally acknowledged questionnaire “International Consultation on Incontinence–Bowel, ICIQ-B”. The ICIQ-B was sent via safe electronic mail system (REDCap) and answered between 02.10.2023 and 09.11.2023 to assess the long-term outcome. One reminder was sent out. The questionnaire asks questions regarding bowel habits and symptoms of anal incontinence and estimates the impact on sexual function and quality of life. Patients are asked both to report on how often they experience said symptom (never – rare – sometimes – usually – always) and how bothersome it is on a numeric rang scale from 0–10 (bother-score). The Danish version of the ICIQ-B has been found to have satisfying reliability and good content, acceptable structural, convergent and discriminant validity [13]. The women also received the St. Marks scoring system [14].

### Literature Review

We performed a PUBMED search in December 2024; ("early secondary repair" OR "early resutur*" OR "early reconstruction") AND (OASI* OR "obstetric anal sphincter injur*" OR "anal sphincter tear" OR "obstetric tear" OR "sphincter injur*) and identified five relevant papers. In addition, a hand search of the references in the retrieved papers was completed, leaving six relevant papers for review.

### Statistics

All variables are summarized as counts (percentages) or median (interquartile range (IQR; Q1–Q3)).

### Ethics and Permits

The study has been approved by the executive board at Herlev University Hospital (No. 21058161) and Center for Regional Development, Capital Region (D-9470260).

## Results

A total of 17 patients had an OASI that needed an early secondary repair within the study period and 11 answered and returned the ICIQ-B, while 10 also answered the St. Marks score. The demographics of the 17 women with early secondary repair are presented in Table 1. Median age at delivery was 33 (21–40), nine women were primiparous and eight were multiparous. All women reported at the initial assessment prior to secondary repair at least one or several symptoms of anal incontinence. The median interval between delivery date and early secondary repair was 16 days (4–27). Overall, the demographics of the subgroup of ICIQ-B respondents do not differ significantly, as shown in Table 1. Among ICIQ-B respondents, the median age was 32 years (21–40), five women were primiparous and six were multiparous. The questionnaires were sent to the patients during fall 2023 resulting in a median follow-up period of 5 years (2.3–5.7), leaving a median age at follow-up of 38 years (23–45).

**Table 1:** Demographic data of the 17 women with early secondary repair

	All (*N* = 17)	ICIQ-B (*N* = 11)
Age at delivery (years)	33 (21–40)	32 (21–40)
Age at follow-up (years)		38 (23–45)
Body mass index (kg/m^2^) (prepregnant)	26 (21–33)	27.5 (24–30)
Parity time of rupture (*n*)		
Primipara	9 (53)	5 (45)
Multipara	8 (47)	6 (55)
Vaginal birth after cesarean (VBAC)	4 (24)	3 (27)
Birthweight (g)	3650 (2875–4506)	3900 (3245–4460)
Perineal rupture (*n*)		
Grade 3A	3 (18)	2 (18)
Grade 3B	7 (41)	5 (45)
Grade 3C	2 (12)	1 (9)
Grade 3, unspecified	1 (6)	1 (9)
Grade 4	3 (18)	1 (9)
Anal sphincter defect verified by ultrasound, unspecified degree	1 (6)	1 (9)
Mediolateral episiotomy	5 (29)	2 (18)
Vacuum assisted delivery	11 (65)	6 (55)
Interval delivery to early repair (days)	16 (4–27)	16 (4–27)
Time from delivery to follow-up (years)		5.1 (2.3–5.7)
Anal incontinence at initial assessment prior to early repair (*n*)		
Fecal incontinence	7 (50)	5 (45)
Fecal urgency	4 (44)	4 (36)
Flatal incontinence	8 (80)	5 (45)
Infection prior to early repair	2 (17)	2 (18)
Antibiotics		
At primary repair	10 (67)	5 (45)
Prior to early repair	8 (50)	5 (45)
At/post early repair	17 (100)	11 (100)
Length after early repair (days)	3 (3–7)	3 (3–3)

Data is presented as median (IQR; Q1–Q3) or counts (%)

### Complications

Seven out of 17 women had no complications (Table 2). Nine women had a postoperative infection. One woman developed a perineal abscess. None had anal fissures. Two women developed recto-vaginal fistulas that needed subsequent surgical treatment. One woman developed an ano-vaginal fistula without need of subsequent surgical treatment. Three women developed vaginoperineal fistulas, that healed spontaneously. None needed a temporary stoma. Eleven had uncomplicated healing after secondary repair. Two women had wound dehiscence during their follow-up period.

**Table 2:** Complications in the 17 women with early secondary repair

	*N* (%)
No complication	7 (41)
Infection	9 (53)
Abscess	1 (6)
Anal fissure	0 (0)
Fistulas	
Ano-/Rectovaginal fistulas*	3 (18)
Vaginoperineal fistulas	3 (18)
Healing process	
Uncomplicated	11 (65)
Delayed healing	6 (35)
Wound dehiscence	2 (12)
Requering re-repair	0 (0)
Requering temporary stoma	0 (0)

Data are presented as number of patients (%)

^*^Two needed subsequent surgical treatment

### Long-Term Outcomes

Ten out of 11 women reported problems with fecal urgency, nine women reported problems with flatal and liquid incontinence, and six women had problems with soiling (Table 3). Six women reported problems with unpredictable bowel accidents and eight women suffered anal/perineal pain. The median St. Marks score was 5.5 (0–11), see Table 4.

**Table 3:** Long-term follow-up using the ICIQ-B in women with early secondary repair (*n* = 11)

	*N* (%)	Bother-score (0–10 points)
Fecal urgency	10 (91)	4 (0–10)
Anal/perineal pain	8 (73)	5.5 (1–8)
Soiling	6 (55)	9.5 (5–10)
Fecal incontinence		
Solid stool	1 (9)	10
Liquid stool	9 (82)	7 (1–10)
Flatal incontinence	9 (82)	8 (2–10)
Bowel accidents without urgency	3 (27)	6 (3–10)
Unpredictable bowel accidents	6 (55)	8 (3–10)
Impact on quality of life		
Restricted sexual function due to anal incontinence	7 (64)	8 (2–10)
Embarrassment	8 (73)	8.5 (4–10)
Keep a lookout for toilets	6 (55)	5 (2–9)
Plan daily activities according to anal incontinence	5 (45)	6 (2–8)
Stay at home	3 (27)	5 (2–9)
Overall interference with everyday life	n/a	3 (1–8)

Data is presented as number of patients (%) or median bother-score points (IQR, Q1-Q3)

**Table 4:** Long-term follow-up using St. Marks score in women with early secondary repair (*n *= 10)

	*N* (%)
Fecal urgency	2 (20)
Fecal incontinence	
Solid stool	3 (30)
Liquid stool	3 (30)
Flatal incontinence	8 (80)
Need to wear a pad	0
Taking constipating medicine	0
Impact on quality of life	6 (60)
St. Marks score	5.5 (0–11)

Data is presented as number of patients (%)

### Quality of Life and Sexual Relations

Eight out of 11 women reported that they were embarrassed by their problems with anal incontinence and six women reported they keep a lookout for toilets when outside of their own homes. Five women planned daily activities to accommodate their anal incontinence and three women stayed at home because of anal incontinence. Seven women reported that their sexual relations were restricted owing to anal incontinence and gave a median bother-score of 8 out of 10 (2–10) in terms of how much it bothers them in their daily life. See Table 3.

### Literature Review

The six previous relevant studies on short- and long-term outcomes after early secondary repair of OASI identified through literary search are presented in Table 5 for comparison. Of the included studies, the follow-up time ranged from 3 months to 6.7 years and included 6–51 patients. The only long-term study with several years of follow-up was the study from Barbosa et al. [10], which also was the largest with 34 respondents at final follow-up.

**Table 5:** Presentation of previous studies on early secondary repair of OASI

Author, study design, country, inclusion period	Population	Complications	Time of final follow-up	Age at follow-up	Outcomes	Questionnaire
Barbosa et al. [10] Retrospective cohort study Denmark 1991–2017	51 included 34 at follow-up (13 of those also included in Sørensen et al.)	Infection 4/51 Abscess 1/51 Fistula 10/51 Re-repair 7/51 Temporary stoma 4/51	*N* = 34 Median 6.7 years (IQR 3.3–16.6)	*N* = 34 Median 40.9 years (IQR 34.2–45.0)	Flatal incontinence 33/34 Liquid incontinence 14/34 Solid incontinence 9/34 Fecal urgency 16/34 Median St Marks score 7 (IQR 2–11) Median Wexner score 3 (IQR 1–9) Impact on QoL 10/34 Dyspareunia 20/34	Wexner Score St. Marks Score FIQL (Fecal Incontinence Quality of Life Scale) Urinary incontinence Sexual dysfunction
Okeahialam et al. [9] Case series United Kingdom 2010–2019	6	Skin dehiscence 3/6 Granulation tissue formation 4/6 “Perineal sinus tract” 2/6	3 months	*N* = 6 29.3 years (SD 8.0)	Asymptomatic 4/6 Fecal urgency 1/6 Fecal urgency + flatal incontinence 1/6 Mean St Marks score 1.6 (SD 3.2)	St Marks score
Lewicky-Gaupp et al. [12] Retrospective chart review USA 2013–2018	18 (7 underwent concurrent repair of rectovaginal fistulas)	Infection 3/18 Fistula 0/18 Re-repair 0/18	3 months	*N* = 18 34.6 years (SD 5.0)	Flatal incontinence 4/18 Fecal incontinence 0/18 Dyspareunia 1/18	Questions regarding accidental loss of liquid or solid stool and difficulty controlling the passage of gas
Arona et al. [15] Case series USA 1991–1994	23 included 17 at follow-up	Superficial separation of perineal skin 5/23 Fistula 1/23	3 months	n/a	Fecal incontinence 0/17 Flatal incontinence 0/17 Dyspareunia 1/15	
Sørensen et al. [11] Case–control Denmark 1991–2005	22 (delayed primary repair and early secondary repair)	Anal fissure 2/22 Abscess 0/22 Fistula 0/22 Re-repair 0/22	50 months (range 2–155)	n/a Age at inclusion 31 (mean) (range 22–38)	Flatal incontinence 14/21 Liquid incontinence 3/21 Solid incontinence 2/21 Fecal urgency 11/21 Mean St. Marks score 6 (range 0–16) Mean Wexner score 4.1 (range 0–13) Affected QoL 8/19	St Marks score Wexner score Quality of Life
Hankins et al. [16] Case series USA 1980–1988	22	Fistula 2/22	12 months	n/a	Asymptomatic 17/19 Anal incontinence 0/19 Dyspareunia 2/19	

## Discussion

We investigated the complications and long-term outcomes after early secondary repair of OASI using a validated questionnaire (ICIQ-B) and found that symptoms with fecal urgency and anal incontinence were highly prevalent. In addition, we found that approximately half of the women had wound complications, where 18% developed ano-/rectovaginal fistulas. Thus, our findings support the previous literature that finds a high risk of symptoms and complications after early secondary repair of OASI [10–12]. Nevertheless, it is important to remember that the included women represent a group with complicated healing, and some would have ended up with a traumatic cloaca, which could severely affect symptoms and quality of life.

Early secondary repair is not a new concept since in 1944 Malpas et al. [17] described its successful employment in six women with complete breakdown of the primary repair within a week of delivery. Early secondary repair (approximately within 21 days after delivery) has been recommended in Denmark since 2008 after Soerensen et al. [11] showed results comparable with late secondary repair. Though such recommendations might still be controversial globally, a handful of studies support these findings although some results indicate that early secondary repair might involve a higher incidence of fistulas [10–12]. Barbosa et al. [10] found fistulas to be more common (19,6%) than in previous studies [11, 12, 15, 16]. In their study, nine out of ten fistulas where ano-/rectovaginal and all required further surgery [10]. We found a similarly high level of ano-/rectovaginal fistulas (18%). The one patient with an anovaginal fistula healed spontaneously, whereas the two patients (12%) with rectovaginal fistulas required subsequent surgery. One of the patients that developed a rectovaginal fistula had a 4th degree OASI which has a known high risk of complications [7]. None of our patients required a temporary stoma which is in contrast to four out of ten patients in the study of Barbosa et al. [10]. None of our patients needed a third repair. As Barbosa et al. [10] suggested, possibly the risk of a fistula is more agreeable for the patients than a disabling perineal wound left to heal and a subsequent late sphincter repair. Though, it is worth considering the risk of temporary stoma when counselling the patients and planning the timing of surgical repair. Overall, late secondary repair has a complication risk of 8–31% [18, 19]. In comparison, in a study of 172 patients with late repair (85% after OASI) Oom et al. found that 23% had post-operative complications—hereof 20% were wound infections, where 21 patients (12%) developed abscesses and 15 (9%) subsequently developed fistulas that all required surgery [20].

In our study, nine out of 17 had postoperative infection after the early secondary repair. After primary repair, a significant occurrence of infection (19%) is seen, and wound dehiscence and wound breakdown > 1 cm is seen in up to 20–25% [7, 8]—probably as a result of decomposing suture materials and tissue due to infection. As such, a high risk of infection and complications is to be expected when operating in a highly contaminated field as in early secondary OASI-repair. In our clinical setting, women with signs of clinical infection (purulent discharge, necrotic tissue or abscess) are treated for their infection with antibiotics and wound debridement when necessary and the early secondary repair is planned when the infection is treated. However, there is no standard treatment with antibiotics or wound debridement in cases with wound dehiscence without clinical signs of infection. This might have an impact on the relatively high risk of wound complications after the early secondary repair at our institution.

Studies on early secondary repair of OASI and anal incontinence are few and small. Most studies report initial outcomes in relation to anal incontinence after 3–6 months, very few report long-term follow-up (4–7 years) and fewer report on quality of life and sexual relations. A recent review by Okeahialam et al. [9] combined five studies on early secondary repair of OASI—a total of 96 patients. Four studies reported short-term follow-up from 3 to 6 months. The only long-term study was also the largest, with 34 patients included at final follow-up, and a median follow-up period of 6.7 years [10]. Anal incontinence symptoms were reported in four studies, most common was flatal incontinence – from 5% after 6 weeks [15], 22% after 3 months [12], 41% after 6 months [11] and 97% after 6 years [10]. Fecal incontinence was reported in two studies with 14% experiencing involuntary leakage of liquid stool at 6 months [11] and 41% at 6 years [10] and for solid stool 9.5% [11] and 26% [10], respectively. This could possibly indicate that anal incontinence-related symptoms increase over time, despite the early secondary repair.

Correspondingly, long-term studies concerning primary repair also find that anal incontinence typically deteriorates [21, 22]. In one study [21] on anal incontinence after primary repair of OASI, 28 out of 31 women with anal incontinence symptoms within the first 3–6 months after delivery were asymptomatic at a 3-year follow-up, but 33 women who did not have anal incontinence symptoms initially, had developed “de novo” symptoms at the 3-year follow-up.

Similarly, in terms of late anal sphincter reconstruction, a deterioration over time has been seen [21, 23–25]. Late anal sphincter reconstruction represents a heterogenous and complex group of women and the procedure is often performed many years after delivery. Generally, studies on long-term results after late anal sphincter reconstruction show high risk of poor outcomes with deterioration in anal incontinence symptoms [19, 23, 24]. Oom et al. found poor outcome at long-term in 40% where almost half of the patients underwent additional surgery for persistent fecal incontinence [20]. In one large study, only 40% maintained fecal continence at a ten year follow-up [24]. In our study, we found both flatal and liquid fecal incontinence reported in nine out of 11 (82%) women at long-term follow-up with a median of 5 years (2.3–5.7), which correlates with the findings of Barbosa et al. [10]. It suggests that anal incontinence symptoms are not constant but fluctuate and possibly deteriorate over time, highlighting the need for long-term follow-up.

Studies on anal incontinence after vaginal delivery without OASI show similar reports on the fluctuation of anal incontinence symptoms and progression over time [26, 27]. One study compared long-term follow-up (> 10 years) on anal incontinence after OASI with two control groups—vaginal delivery with no OASI and caesarean section and found that 53% of the women with OASI reported anal incontinence symptoms in contrast to 19% and 11% in the control groups, respectively [28]. Although anal incontinence can be a natural consequence of vaginal delivery, it is still more common in women with OASI [22, 26, 27]. Nilsson et al. found a three- and fivefold increase in fecal incontinence after one or two OASI, respectively, and found that at the age of 60 years, less than one in five women with previous OASI was continent [27]. Anal incontinence issues in women are probably underestimated and underreported in general.

Of women with OASI, those with the need of secondary repair represent a complex group due to previous surgery complicated by infection and are expected to have more frequent and severe complications as well as a higher risk of symptoms both short- and long-term, compared to those with uncomplicated wound healing after OASI. Our findings support this.

### Quality of Life

As shown in previous studies on OASI, anal incontinence undoubtedly impacts quality of life [28, 29]. In the study of Barbosa et al. on early secondary repair anal incontinence was found to have some or major impact on quality of life in 29% [10]. In our study, almost a third reported problems with bowel accidents without previous urgency and half of the women had experienced unpredictable bowel accidents. Such findings had a major impact on the women’s daily life. Thus, we found problems with anal incontinence to be associated with embarrassment in almost two thirds, with a high impact on daily life (median of 8.5 on the bother-score). Almost half of the respondents altered their life to some extent, some even daily, to accommodate the disability anal incontinence issues entail and reported a considerable impact on daily life (median of six on the bother-score). A third reported that they sometimes avoided leaving their home because of anal incontinence. Interestingly, the indication of overall interference with everyday life was relatively low (median of 3 on the bother-score, ranging from 1–8), suggesting that most of the women had adapted to their situation using different coping strategies and precautions in their daily life.

### Sexual Functions

We found that two thirds reported affected sexual function due to anal incontinence and gave a high median score of 8 out of 10 on the bother-score, indicating that anal incontinence and the concurrent problems have a high impact on sexual relations. This is indeed noticeable as it was mainly premenopausal women with a median age at follow-up of 38 years (23–45). OASI has been shown to be the strongest predictor for postponed coital onset after delivery [30]. Barbosa et al. found a reduction in sexual desire in 53% of women with early secondary repair at long-term follow-up, 59% had experienced dyspareunia in the past 2 months and 21% had the sensation of a narrowed vagina after the repair [10]. Mous et al. concluded that OASI is an important risk factor for both fecal incontinence and sexual complaints even more than two decades after delivery [26].

### Significance

Traditionally, secondary OASI-repair has been performed more than 3 to 6 months after delivery—often many years after the delivery with an OASI. The time until the secondary repair is typically complicated with symptoms of anal incontinence that affects quality of life and concerns about long-term outcomes. Early secondary repair is based on few studies with a limited number of patients. Our findings support that women requiring early secondary repair have a high risk of long-term symptoms, despite treatment with secondary repair in a highly specialized unit within the first 21 days after delivery. Thus, this group represents a complex group with high risk of complications and the procedure with early secondary repair can only be recommended in cases with severe wound dehiscence and/or infection.

### Strengths and Limitations

One of the strengths of this long-term follow-up study is the use of the validated and recommended ICIQ-B questionnaire [13]. The validity of the complications is high as the medical journals from the regional hospitals, where it is reported by the treating physician in detail, were thoroughly revised. However, minor complications may have been overlooked if treated at the patient’s general practitioner without our knowledge. The early secondary sphincter repair was, in all cases, performed by experienced specialists in urogynecology, making the treatment standardized.

Our study is limited by a retrospective design with a cohort from a single institution, the basis of self-reported data and no baseline anal physiological testing preoperatively. The small sample size and the lack of a control group limits us to report descriptive results and excludes firm statistical conclusions. It is a limitation that no standardized questionnaire was used prior to or shortly after the early secondary repair and, thus, comparison of symptoms before and after repair and at short- and long-term follow-up was not possible.

### Future Studies

Larger sample size would enable identification of risk factors of complicated healing. A prospective design with several follow-up controls at different time-points using the same validated questionnaire would increase our understanding of the development of symptoms and bother and whether these factors fluctuate over time. The significant impact on quality of life and sexual relations as found in these young women calls for further studies focusing on perineal pain or altered sensibility, and risk of soiling during sexual activity.

In conclusion, early secondary repair of OASI in women with severe wound dehiscence involving the anal sphincter may be necessary. However, this group have a high risk of anal incontinence at long-term. Nevertheless, the early secondary repair might be an acceptable alternative to late anal sphincter reconstruction, reducing the waiting time and symptoms related to the wound dehiscence of the anal sphincter complex. We encourage more long-term follow-up studies using standardized assessment of complications and symptoms.

## Abbreviations

ICD-10: The International Classification of Diseases and Related Health Problems
ICIQ-B: International Consultation on Incontinence Questionnaire-Bowel
IQR: Interquartile range
OASI: Obstetric anal sphincter injury
QoL: Quality of Life
